# Annotations

**Published:** 1903-04-11

**Authors:** 


### Annotations.

### The Municipal Slaughterhouse.

In a certain suburb there is a butcher's shop on which is inscribed in bold letters the announcement "Licensed Slaughter-house on the Premises." It must be presumed that the owner of that shop thinks that it will commend his meat to his customers to know that the animals are slaughtered on the premises. Doubtless the haters of foreign meat find a gloomy satisfaction in the spectacle when they see an ox or half-a-dozen sheep enter the portals of the slaughter-house. It behoves us not to ask if the butcher who ostentatiously kills one ox in sight of the world may not also buy frozen meat; the purchasers at least believe that they are getting home-fed and home-killed meat. So it may work out to the profit of the butcher. But surely the existence of a slaughter-house in a busy street in a crowded neighbourhood is an offence. The slaying of animals for food is not, at the best, a rose-water business, but it should at least be carried out with as little cruelty and as much cleanliness as possible. There is no guarantee for either of these points in a private slaughter-house. All may be right when the license is granted, but what surety is there that a high standard is kept up afterwards? The slaughter-house should be under regular supervision both as to the cleanliness of the place and the arrangements for killing, and a properly qualified veterinary surgeon should examine every carcase before it is allowed to leave the place, in order to make sure that it is fit for human food. Without such guarantees it is impossible to prevent the sale of diseased meat. A reaction seems to be setting in against municipal trading, but in all matters of health the municipality must interfere to secure the welfare of the community as a whole, which in some cases does not coincide with the interest of individuals. There is no reason to prevent a butcher buying his animals alive wherever he sees fit, but before they are offered for food they ought to be examined by an expert whose position raises him above the necessity of considering individual interests, and as this can be attained only by a municipal authority, the work can be best carried out in a municipal slaughter-house.

### Compliments.

At an inquest in Kensington last week the profession was the recipient of one of those left-handed attacks to which it has grown more or less accustomed and which are really right-handed compliments. The inquiry was one into the circumstances attending the death of a newly-born child, and indirectly its fate was ascribed to the "heartlessness" of the profession. There were no difficulties attending its birth, but it appears that when the infant arrived upon the scene there were three other children in the house suffering from whooping cough, and only the father at hand to attend to either them or the mother. The parish doctor duly arrived when summoned, but it appears that before this the father thought fit to knock up three general practitioners in succession, who for one reason and another declined to accept the responsibility of a confinement at five minutes' notice in the middle of the night. Hence the "heartlessness" of the profession, and hence these tears. In reality, however, this sort of indirect charge is a complimentary acknowledgment that members of the medical profession are in a general way very benevolent people, and ready to do a great deal for nothing out of pure goodwill toward their fellow men. It is a very pretty reputation in itself, but not one aimed at by any other class of men who live on the fruits of their own labour, and is certainly not one that medical men in these days are in any way called on to keep up. In the days when "clubs" were not, and the Poor-law service was a very imperfectly organised piece of machinery, there was some reason why medical men should occasionally step into the breach. Nowadays, however, the organisation of State, charitable, and provident medical relief is, in London at any rate, so complete and minute, that any woman who goes unattended in her confinement has only the carelessness of herself or her friends to blame.

### Realism.

Though Art, with a capital A, is eternal, the terms which its votaries apply to it vary so often that we cannot be certain that "Realism" has today the same meaning that it had in the days when The Hospital was young. However this may be, a picture has lately attracted our attention which proves that "The Realistic School," as we understand it, existed long before the birth of the young man who was the first to invent and proudly christen it. It is a picture by Nicholas Maas, painted in the seventeenth century, and represents a number of Dutch peasant women bathing from a barge on one of the many waterways of Holland. The picture is full of life, and the figures are depicted in various stages of undress, but anything less like the pretty pictures of the human form divine to which we are accustomed in the Academy, and more like the same form as we see it in the course of our daily work, it is difficult to imagine. It is not, we should think, a work likely to be acquired by anyone on the look out for a drawing-room decoration, but to a man of medical training its truthful unattractiveness as a picture will be outweighed by its attractive truth as a representation of the human figure as commonly seen by him. It will be found in the window of an art dealer at the Piccadilly end of Bond Street, and it is well worth a stroll to go and see it. In the same connection any medical man who possesses or has access to a set of Hogarth's "Marriage à la Mode" should look at the fifth plate in the light of a criticism of it by William Hazlitt. This plate depicts the Bagnio scene, and Hazlitt, otherwise a great admirer of Hogarth's genius, condemned it entirely, on the ground that the attitude of the wounded husband is an impossible one for any man to assume whether standing or falling. This, if not a purely medical criticism, is at least one the truth of which medical men are well fitted to judge. To ourselves it seems that Hogarth as usual was right and Hazlitt wrong. Where collapse is occurring from sudden heart failure or the shock of a wound through the body, it seems natural that the tension of the extensor muscles of the arm should be preserved momentarily longer than that of the muscles of the thigh which are so much further away from the cerebral Motor area.

